# Advantages of FDM and gamma irradiation to manufacture personalized medical devices for airway obstructions

**DOI:** 10.3389/fbioe.2023.1148295

**Published:** 2023-06-30

**Authors:** Beatriz Aráoz, Gastón Bellía-Munzón, Juan I. Bousquet, Élida B. Hermida

**Affiliations:** ^1^ Laboratory of Biomaterials, Biomechanics, and Bioinstrumentation (Lab3Bio), Instituto de Tecnologías Emergentes y Ciencias Aplicadas (ITECA), UNSAM-CONICET, Escuela de Ciencia y Tecnología, Buenos Aires, Argentina; ^2^ Hospital General de Niños “Pedro de Elizalde”, Buenos Aires, Argentina

**Keywords:** additive manufacturing, airway, case report, bronchomalacia, splint, polycaprolactone

## Abstract

In the early childhood population, congenital airway conditions like bronchomalacia (BM) can pose a life-threatening threat. A breakthrough technology called additive manufacturing, or 3D printing, makes it feasible to create a biomedical device that aids in the treatment of airway obstruction. This article describes how a polycaprolactone (PCL) splint for the upper airways can be created using the fusion deposition technique (FDM) and sterilized using gamma radiation. It is presented as a simple, accessible, and cost-reduced alternative that complements other techniques using more expensive and sophisticated printing methods. Thermomechanical and morphological analysis proved that FDM and sterilizing by gamma irradiation are both appropriate methods for producing splints to treat life-threatening airway blockages. Additionally, the 3D-printed splints’ effectiveness in treating a young patient with BM that was life-threatening was assessed by medical professionals. In this regard, the case report of a patient with 34 months of follow-up is presented. Splints manufactured by this affordable 3D printing method successfully surpass breathing arrest in life-threatening airway obstruction in pediatric patients. The success of this procedure represents a fundamental contribution to the treatment of the population in countries where access to expensive and complex technologies is not available.

## 1 Introduction

Airway congenital problems such as laryngotracheal cleft, tracheal stenosis, tracheomalacia (TM), bronchomalacia (BM), and tracheobronchomalacia (TBM) are often life-threatening in the early childhood population. Over the last decades, imaging techniques have improved diagnosis and innovative management techniques are leading to a much better prognosis for children with these diseases. For example, 3D printing, also known as additive manufacturing or rapid prototyping, is one such innovative technique, making it possible to build a biomedical device that helps resolve airway obstruction. The biomedical device is made according to the patient, since the shape and size are derived from digital images of the reduced lumen (CT, MRI, 3D ultrasound).

Recently Stramiello et al. (2020) presented a review to identify the current role of 3D printing in pediatric airway obstructions: 5 articles reported direct interventions in human cases where external splints were applied to children with TBM, reducing airway symptoms, and the largest case series published by Les et al. (2019) with 15 pediatric patients, where 3D print external splints allowed an improvement in ventilatory requirements in the majority of patients.

These promising results were achieved using splints printed from PCL powder using selective laser sintering (SLS) (Jamróz et al., 2018). The SLS technology fused together fine particles of powdered materials like plastic or ceramics, using a powerful laser beam; it is useful when printing complex objects with fine details, such as implants, fixation devices, etc.

However, the cost of an SLS machine is quite high when compared to printers based on the fused deposition method (FDM) of thermoplastic filaments. In fact, currently FDM systems are at least two orders of magnitude cheaper than SLS (Prusa Research MK2 printer vs. SLS EOS P 100 Formiga system, for instance), not including local fees, maintenance or trained operators.

Spare parts for the FDM printer are less expensive and easier to locate on the marketplaces of periphery nations, which is a key economic feature. FDM printers uses a thermal resistor at the hot end as the heat source to melt the polymer, whereas an SLS printer uses a laser, which is much more expensive. Considering the time to produce the splints, and the small number required for the surgery, FDM takes longer than SLS printers, but a splint is finished in a day. Additionally, during an SLS printing process, at least one-third of the powder reservoir volume must remain full although only part of this powder is used to create the splint, whereas FDM uses just the filament required to print the splint. Given the high cost of biomaterials for biomedical applications, this trait is crucial. Thus, FDM is preferable over SLS in rare and small-scale production.

Due to the variability of the manufacturing processes, there is no possibility of giving one universal set of guidelines for both printing methods. For example, SLS and FDM require different starting materials—powder for SLS and filament for FDM—. Both have different process parameters related to the printing temperature: laser beam energy and powder bed temperature for SLS and extrusion and printing platform temperature for FDM (Bourell et al., 2014; Kozak et al., 2021; Montez et al., 2022). In addition to these differences, the main driving force to study the possibility of printing splints to mitigate or resolve pediatric airway obstructions is to achieve an affordable manufacturing process mainly for underdeveloped countries (Kumar, Kumar, and Chohan, 2021).

Huang et al. (2016) presented a PCL splint printed by FDM that was used to treat a 46-year old woman with TM. The patient was discharged 2 weeks after surgery and followed up for 3 months without observing adverse effects. The splint could withstand the forces exerted on it, but the splint was not characterized mechanically nor physicochemically. Also, the sterilization technique was not indicated. Thus, this work details how a splint for upper airways can be manufactured by FDM and sterilized using γ-irradiation. The printed devices were tested to determine the dimensional accuracy, whether processing temperatures or irradiation dose introduced any thermo-mechanical change to the material and, if the requirements stated by Hollister et al. were achieved: 1) less than 10% splint displacement under 20 N and 2) greater than 20% displacement under 15 N opening load (Hollister et al., 2015). In addition, 3D printed splints were evaluated by a medical team for application in a pediatric patient with life-threatening BM. In this regard, the clinical case of a patient with 34 months of follow-up is presented.

## 2 Materials and methods

### 2.1 Study design

A pediatric patient, evaluated by the medical board, was approved as a candidate for life-supporting and life-enhancing treatment of bronchomalacia. A PCL splint was designed according to the patient’s anatomy, printed by FDM, sterilized by gamma irradiation and implanted. The thermal and mechanical characterization of the splint was measured before and after irradiation. The patient was followed up for 34 months.

### 2.2 Materials

The filament used to print the splints was composed of polycaprolactone (PCL, medical research quality, Caproprene™ 100 M, from Poly-Med Inc., M_n_: 89 kDa (Mn) and M_w_: 147 kDa). Solvents used (acetone, ethanol) were analytical grade.

### 2.3 Splint design and 3D printing

Splints were designed to exactly fit the patient’s anatomy and keep the bronchus open. Briefly, CT scans were taken from the patient’s airways, processed, and splints were manufactured according to the mechanical design inputs (SQ1–SQ4) proposed by Hollister et al. (2015) *.* The internal diameter and length of the splint were based on the reconstructed malacic airways. A reconstruction of the patient’s airways was printed using stereolithography (SLA) from a computed tomography model to verify the splint design and plan the surgery (Supplementary Figure S4). Splint internal diameter and length were customised to the reconstructed airways. According to work in which similar splints have been used, the wall thickness can vary between 1 mm and 3 mm (Hollister et al., 2018; Ramaraju et al., 2022; Yu et al., 2022). The designed splints were 14.0 mm in length (L), 1.5 and 2.0 mm of wall thickness (T), and 8.0 and 10.0 mm of inner diameter (D). The 14.0 × 2.0 × 8.0 mm (LxTxD) splint, referred as model 1, was used for patient’s surgery and the splint of 14.0 × 1.5 × 10.0 mm, referred as model 2, was used to compare the mechanical properties of the implanted splint with the one that would meet the minimum mechanical requirements. The surgeon chose to use 2.0 mm thick splints at the time of the surgery. An open-source direct extrusion FDM 3D printer (PRUSA research Model i3 MK2) was adequate to gain control of the printing parameters and achieve excellent print quality. The software used to control the printer parameters was Slic3r PRUSA i3 MK2S version 0.8.3. The material composition of all the 3D printer parts in contact with the filament (nozzle, hot-end, barrel, and extruder) were stainless steel and Teflon^®^. Parts were mechanically cleaned first and then with serial solvents: acetone, ethanol, 70% v/v ethanol. Medical research grade PCL was used to ensure cleanliness by melting 2 m of material through the hot-end. The 3D printer was placed in a clean area inside a horizontal laminar flow hood (CASIBA HL) (Supplementary Figure S1). The internal diameter of the nozzle was 0.3 mm. The printing parameters were adjusted to improve: 1. contact among printed layers, 2. printing quality of the suture holes, 3. reduction of oozing (surface finishing), and 4. mechanical properties of splints. These main parameters were the printing temperature, the infill pattern, the printing speed, and the number of perimeters. The printing time per splint was ca. 30 min, all printed in the same place of the printer bed and with the printer mounted in the laminar bench. The hot-end was preheated and the filament was loaded into the printer at 90°C and the printing temperature set at 100 °C. The layer height was set at 0.2 mm. Splints were printed at 6 mm/s and the structures built performing a rectilinear pattern with 100% infill. To improve the adhesion of the structure to the bed and to avoid any contamination from the bed, each splint was built onto a raft printed with the same filament. Conditions to build the raft were carefully selected to achieve a stable 3D-printed splint easily detachable from the raft. The orientation of the object on the printer bed was defined according to the way that the surgeon performs the sutures to hold the splint to the bronchus, that is, between consecutive holes in the z direction of the splint, as shown in Supplementary Figure S2A. In fact, the splint was printed in a vertical position on the printer bed, so that the sutures will contribute to keep the layers together, in contrast to the case proposed in Supplementary Figure S2B. After 3D printing no additional post-processing steps regarding cleaning to remove residual debris, cutting, or polishing were needed.

Splint dimensions (length, inner diameter and wall thickness) were measured using a digital caliper repeating the process at least 20 times for each dimension and repeated in six 3D printed splints of model 1. Layer thickness was determined from the SEM images, using ImageJ^®^ software (Schneider et al., 2012).

### 2.4 Splint post-processing

Cleaned splints were arranged in a double sealed package and sterilized by gamma irradiation; a dose of 35 kGy at a dose rate of 9.5 kGy/h was applied at the Semi-Industrial Irradiation Plant of the National Atomic Energy Commission in Argentina. The applied dose eliminates microorganisms, without compromising the material properties and the biocompatibility (Augustine et al., 2015).

### 2.5 Filament and splint morphological and physicochemical testing

#### 2.5.1 µCT scans

To evaluate dimensional discrepancies between modeled and 3D printed splints microcomputed tomography images were acquired in a µCT (SkyScan 1,173, Bruker) at 60 kV and 50 µA with a resolution of 10.0 µm voxel size. DICOM images of splints were post-processed into ImageJ (Fiji) software (Schindelin et al., 2012). Masks were created to calculate volume discrepancies in the splints.

#### 2.5.2 Optical and electronic microscopies

To evaluate surface morphology, contact between layers, and suture holes quality, images of splints were acquired with a 60x microscope and with a scanning electron microscope (FEI Quanta 250, equipped with a low vacuum detector) without any pretreatment of the splints.

#### 2.5.3 Differential scanning calorimetry (DSC)

ca. 10 mg PCL filaments or splints were placed in an aluminum pan and loaded into a simultaneous thermal analysis equipment DSC-DTA-TG (TA Instrument SDT Q600) and ramped 10°C/min from 25°C to 600°C. TA software was used to determine the peak temperature at the melt transition (T_m_), and the enthalpy of fusion (ΔH_f_). Crystallinity was calculated on considering the enthalpy of fusion of a 100% crystalline PCL (139.5 J/g) as previously reported (Pitt et al., 1981).

#### 2.5.4 Fourier-transform infrared spectroscopy (FTIR)

The chemical characterization of functional groups was evaluated by direct analysis of FTIR-ATR, setting the samples on ZnSe crystal. Infrared spectra were obtained on a FTIR Nicolet™ iS20 FTIR Spectrometer (Thermo Scientific) with 16 scans and a resolution of 4 cm^-1^.

### 2.6 Splint mechanical testing

Mechanical properties of pristine and irradiated 3D printed splints were measured to evaluate the mechanical response in terms of the requirements previously established by Hollister et al.: firstly, the splint should be stiff enough to maintain the airway opened ―once the splint is sutured to the airway, forces are imposed on it and the splint should prevents re-collapse of the airway walls, therefore, allowing breathing. Secondly, the splint should allow normal growth of the airway ―once the splint is implanted and degrading airways will expand during growth, therefore, the splint should be flexible enough to accompany airway expansion not limiting the air flow through an estimated *in vivo* degradation time of ca. 3 years for PCL (Sun et al., 2006; Siddiqui et al., 2018). Compression and opening tests were performed in a DMA Q800 (TA Instruments), using a compression clamp of 40 mm in diameter and film tension clamp adapted with acrylic hooks (Figures 3A,B) and a displacement ramp: 3 mm/min. For the opening tests splints 3D printed splints were cut in 3 segments minimizing the strength applied to them using a cutter. Cuts were performed in between printed layers and preferably throughout the middle of suture holes, thus the segments obtained contained one set of aligned suture holes. The length of splints was ca. 14 mm. Supplementary Figure S3A shows the splint model 1 and the scheme where the cuts were performed. Supplementary Figure S3B,C, show the segments B and C obtained after the cut. The larger and shorter widths of segments are shown in both schemes and the distances were measured with a digital caliper. Experiments were performed in triplicate or in sextuplicate according to the number of samples.

### 2.7 Medical evaluation of the patient

Laryngotracheobronchoscopies were performed preoperative and at 19 days, 6 months, and 34 months postoperative. Images were acquired with a Jackson videolaryngoscope with 2.8 mm and 0-degree Karl Storz optics for initial diagnosis. For post-surgical follow-up, a 4 mm Storz optic was used. Computational tomography (CT) was performed in a Siemens helicoidal axial tomography, SOMATOM Emotion 16, with 16 slices.

### 2.8 Statistical methods

The results are presented as the mean S.D. ANOVA (analysis of variance) and a Student’s t-test were carried out to determine the significant differences among the groups. The observed differences were statistically significant when *p* < 0.05.

## 3 Results

### 3.1 Splint geometric analysis

**FIGURE 1:**
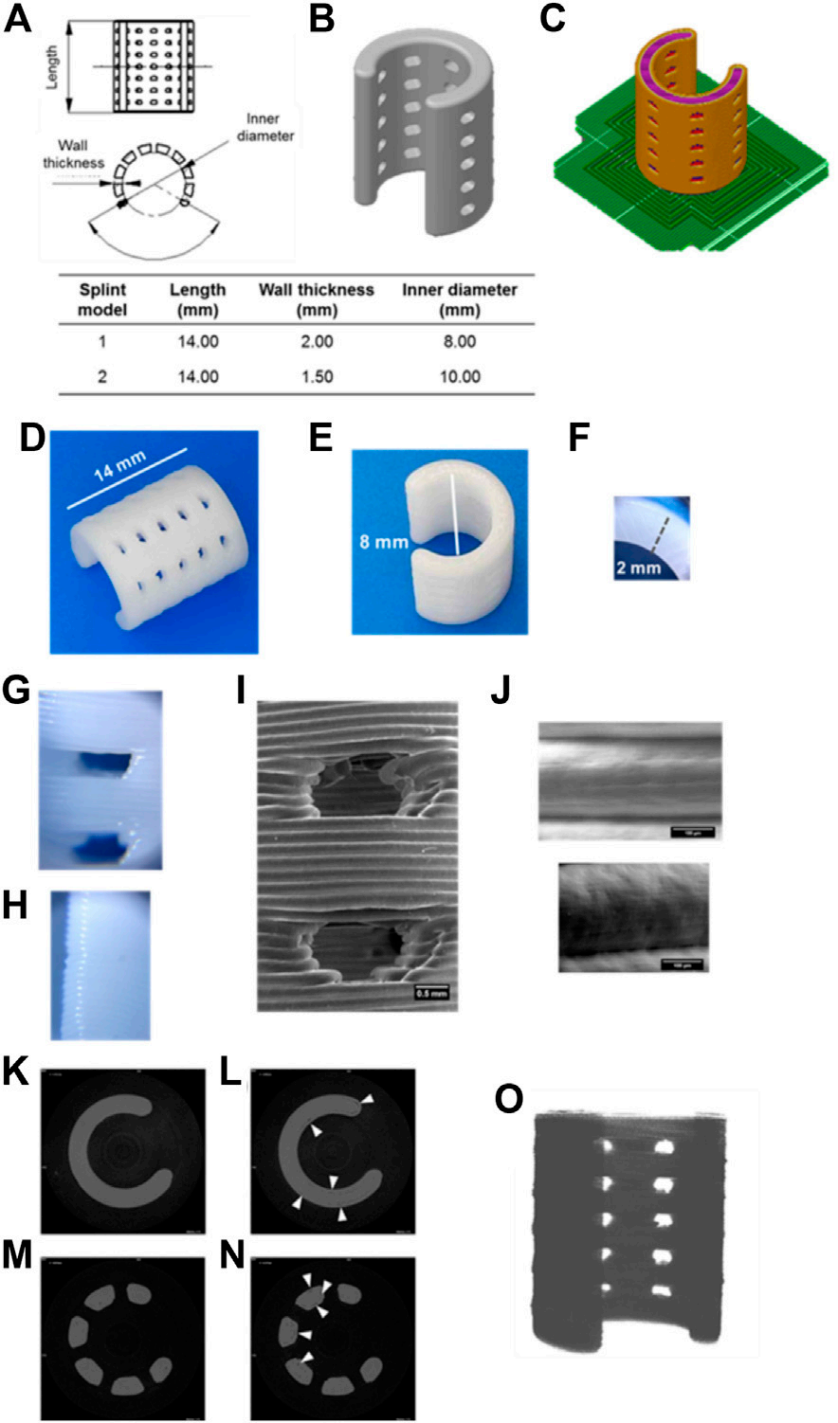
Printing quality of splints produced by FDM evaluated by optical, electronic, and micro-CT images. Length, wall thickness and inner diameter of the modeled splints **(A–B)**. Splint position and printing pattern, raft is denoted in green color **(C)**. Splint model 1 printed by FDM **(D–F)**. Optical microscopy images of suture holes and long ends of the 3D printed splint (×60 magnification) **(G–H)**. Electronic microscopy images of suture holes **(I)** and contact between layers **(J)**. Micro-tomography images of continuous s **(K–L)** and suture hole **(M–N)** of splints (7.5 µm scanned layer height). White arrows indicate interstitial discontinuities **(L, N)**. Micro-tomography reconstruction of the 3D printed splint **(O)**.

The printing quality was assessed by comparing the splint dimensions to the digital model, observing the contact between layers and the presence of inner pores. Figure 1 depicts the assessment of the splint model 1 by optical and electronic micrography as well as by microcomputed tomography. The characteristic dimensions of the printed device (Figures 1D,F) agreed with the ones of the digital model (Figures 1A,B) (see Splint design in 2). The measured length, inner diameter, and wall thickness of splint model 1 were 14.06 ± 0.03 mm, 7.99 ± 0.01 mm, and 2.03 ± 0.01 mm respectively. Electronic micrographs (Figures 1I,J) show good layer-to-layer contact with proper material continuity. No bubbles or micro-voids were observed within layers. The layer thickness was 0.195 ± 0.013 mm. Micro-CT scan shows good internal continuity within the splint (Figures 1K,M), and some micro-voids were observed within the 3D printed splint, Figures 1L,N. Computational analysis was applied to evaluate the discrepancies between the model and the splint. The void volume discrepancy was quantitatively determined by calculating the difference between the volume of the micro-CT reconstruction of the 3D printed splint (Figure 1O) and the reconstructed solid image. Volume voids discrepancies were below 5%.

### 3.2 Splint physicochemical characterization

**FIGURE 2:**
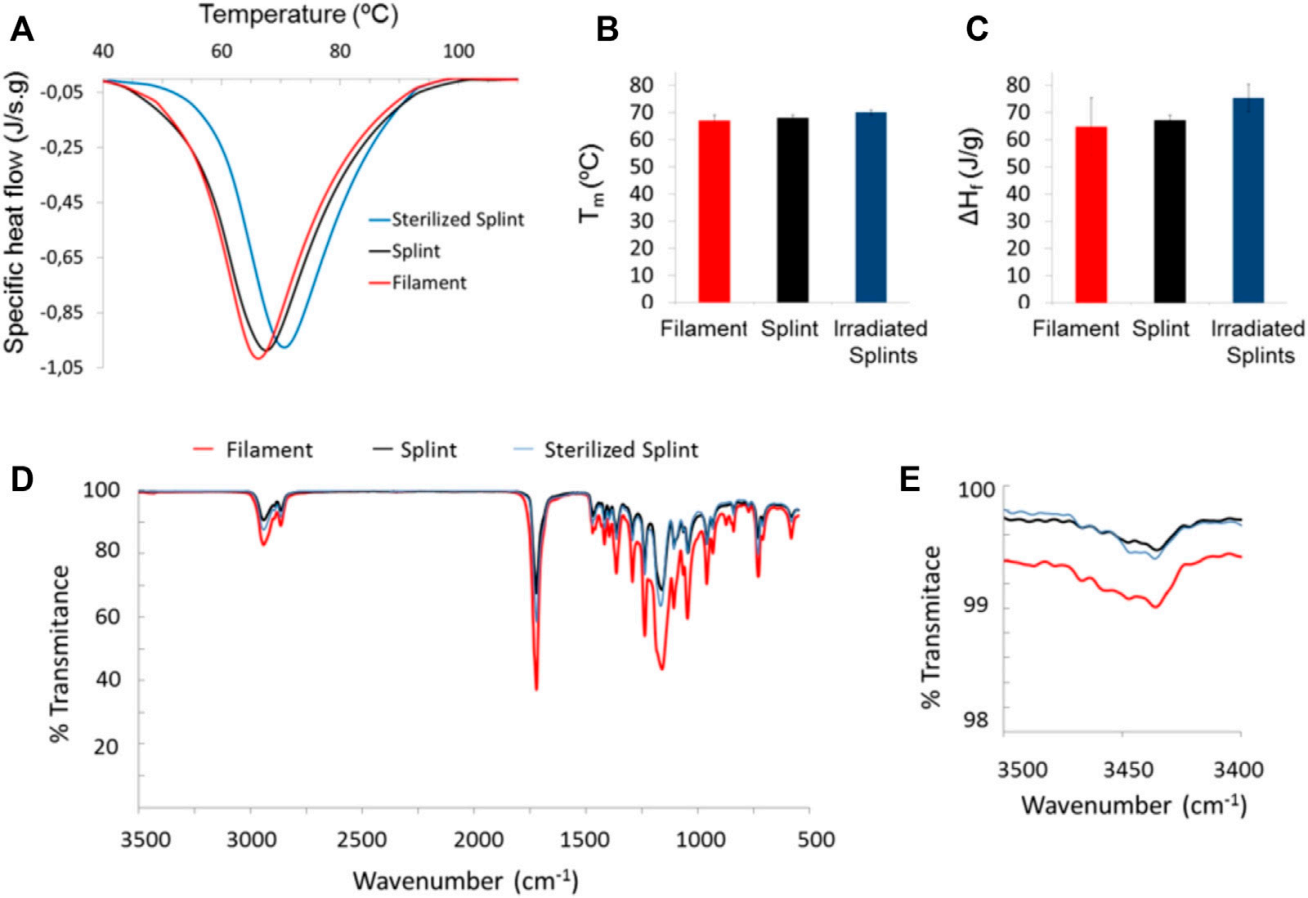
DSC and FTIR of the PCL filament as received and splints pre- and post-irradiation: DSC thermograms **(A)**, melt transition temperature, T_m_, and fusion enthalpy, ΔH_f_, [**(B)** and **(C)**, respectively]; FTIR spectra of filament and 3D printed splints before and after sterilization **(D)**, and zoom of a FTIR wavelength segment **(E)**.

Characterization of splints after 3D printing and γ-irradiation was evaluated by DSC and FTIR (Figure 2). Melt transition changes due to printing and sterilization are presented in Figure 2A. The calculated crystallinity degree was 0.46 ± 0.08 for the filament, 0.48 ± 0.01 for the splint, and 0.54 ± 0.04 for the sterilized splint. The melting temperature of PCL did not change after 3D printing (Figure 2B), within the experimental error, but the enthalpy of fusion (Figure 2C) and thus the degree of crystallinity, slightly increases. γ-irradiation produces a small increase both in the melting point and the degree of enthalpy of fusion (Figures 2A–C). Figure 2D shows FTIR spectra of filament, splints, and gamma irradiated splits; there were no changes in the characteristic spectra of PCL (Elzein et al., 2004): a peak at 1720 cm^-1^ due to carbonyl stretching and a peak at 1,158 cm^-1^ due to C-O and C-C stretching. Peaks between 2,864 cm^-1^ and 2,942 cm^-1^ correspond to CH_2_ stretching. Figure 2E presents a magnification of FTIR spectra around 3,450 cm^-1^ where a very low transmittance band is present in the filament and splints before and after irradiation. Differences in intensity are a result of the clamping pressure and were not assessed.

### 3.3 Splint mechanical response

**FIGURE 3:**
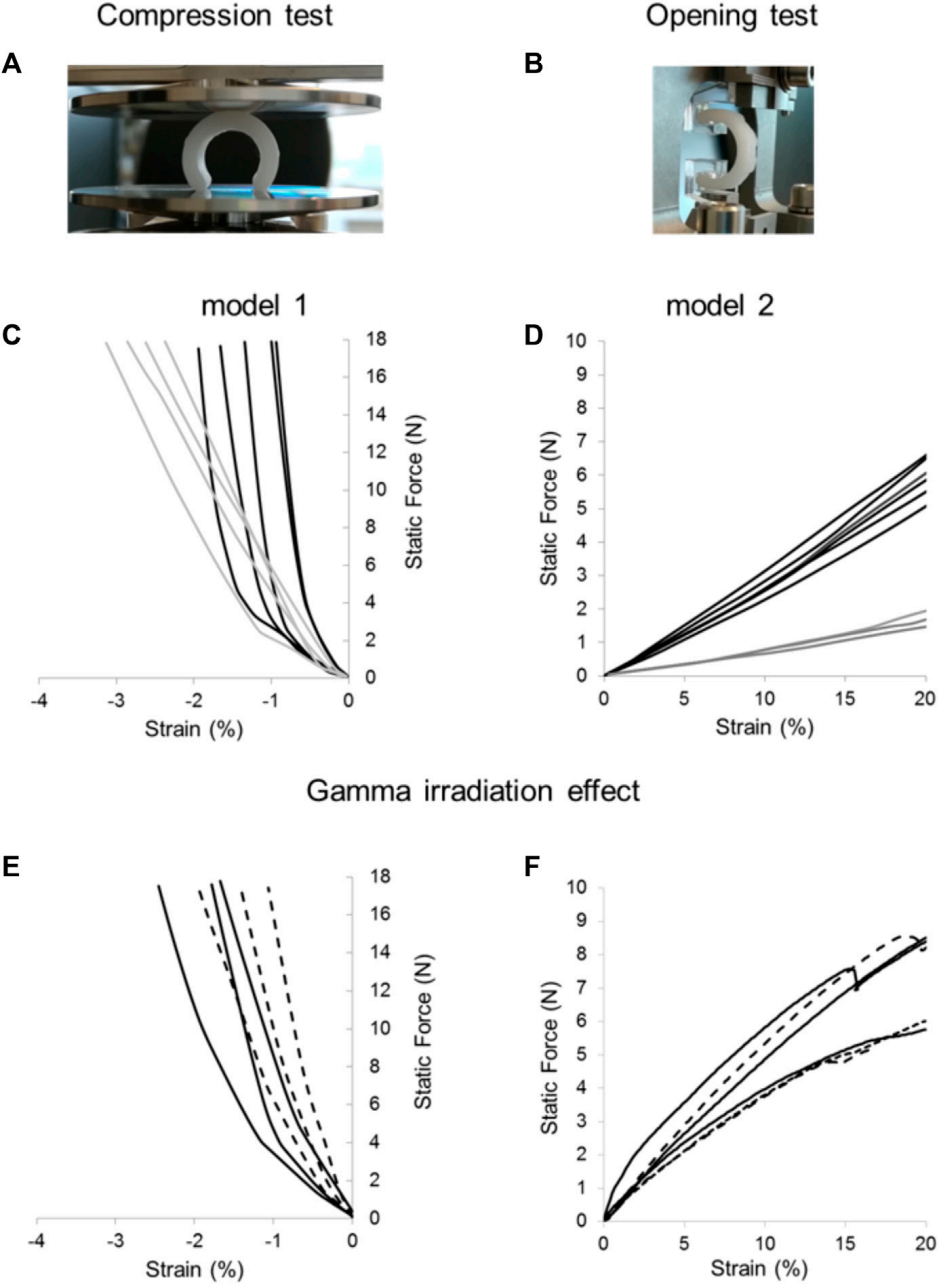
Compression and opening tests of FDM 3D printed splints. Samples were mounted between the DMA clamps for compression **(A)** and opening **(B)** tests. Dispersion of mechanical properties within replicas of 3D printed splints: compression **(C)** and opening **(D)** tests performed on model 1 (black lines) and model 2 (grey lines). Effect of gamma irradiation on mechanical properties of splints for compression **(E)** and opening **(F)** tests before (continuous line) and after gamma irradiation (dashed line). Curves with the same color represent replicas of the splints evaluated mechanically under the same conditions to highlight experimental dispersion of data.

Splints were designed varying the internal diameter and the thickness of the printed wall being model 1 the one with the thicker wall and model 2 the one with the higher internal diameter. Figure 3 shows the mechanical behavior of splints before and after sterilization. Compression curves of models 1 and 2 show a displacement lower than 3.5% at 18 N; under 10 N opening load a displacement greater than 20% is seen. The dispersion in the mechanical response of replicas of model 1 and model 2 is ± 0.5% for the displacement in compression (Figure 3C) and lower than ± 1 N load in tension at 20% displacement, Figure 3D. The effect of sterilization on the mechanical response of splints was evaluated and shown in Figures 3E,F, for both compression and opening tests. Differences in the curves between irradiated and pristine samples are comparable to the dispersion of curves between replicas of the same model. It is important to consider that the splints were cut into 3 segments for the opening tests, using a sharp cutter to ensure adequate subjection with the DMA clamps, which have a width of barely 8 mm. As a result, segments were examined to see if under 5 N (iso-strain condition), one-third of the splint moved by at least 20%. Within the experimental error, each had the identical mechanical response, which supports the iso-strain condition assumption (Chawla, 2019).

### 3.4 Clinical history of pediatric patient with life-threatening congenital tracheal stenosis and left bronchomalacia

**FIGURE 4:**
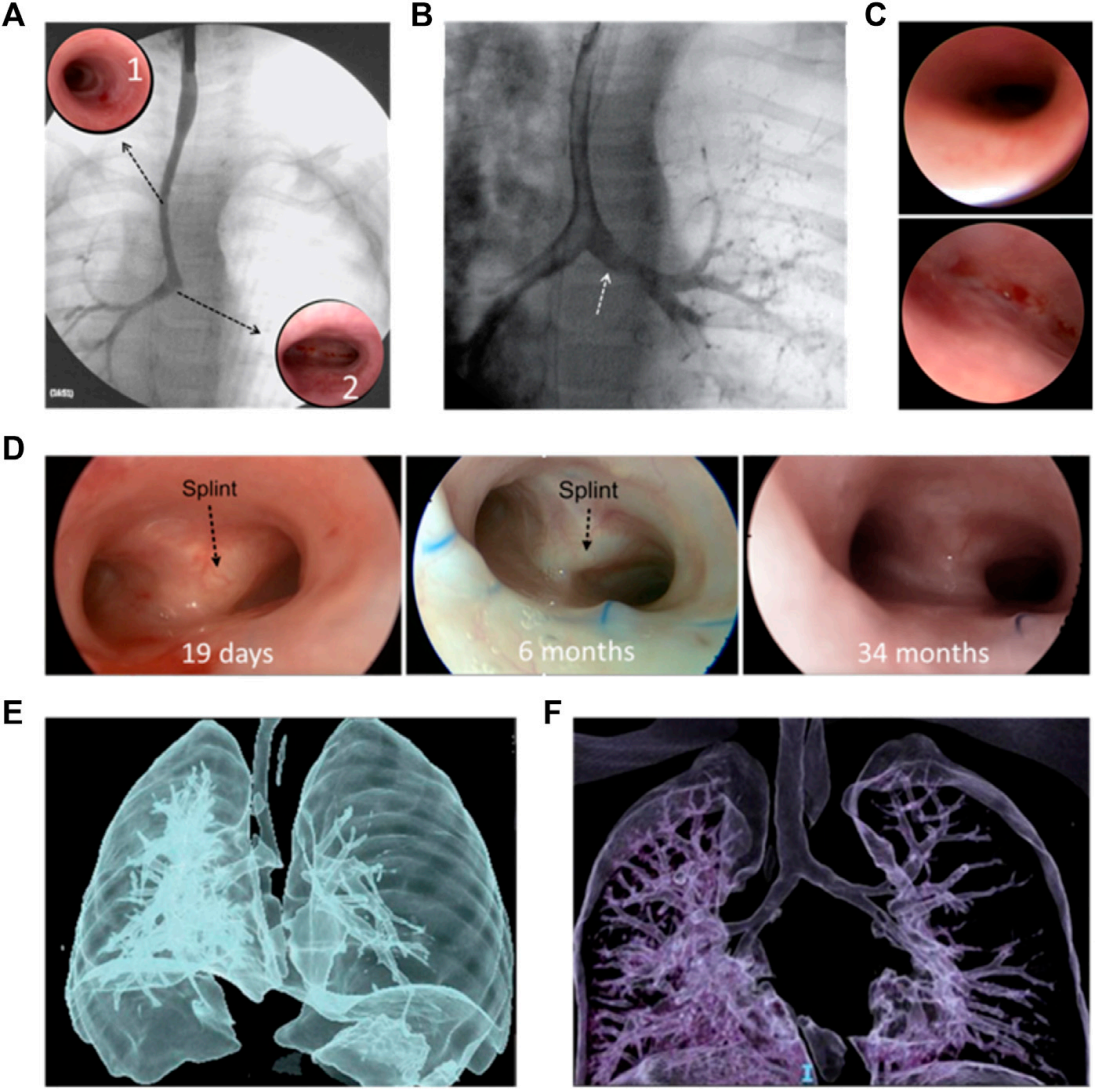
Clinical evaluation of a patient with BM pre- and post-operative: Pre-operative: **(A)** tracheobronchography with long tracheal stenosis, good caliper right bronchus, and absence of contrast passage to the left bronchus. Complete stenotic tracheal rings (**A**1); normal carina and origin of the right bronchus (**A**2). **(B)** With pressure inside the airway, passage to the left bronchus (white arrow). **(C)** right bronchus of normal diameter (top) and Malacic left bronchus (bottom), without a lumen. **(D)** Post-operative: Good bronchial lumen 19 days (left) and 6 months (middle) after surgery; black arrows indicate the splint observed through the mucosa. 34 months after the operation no splint was observed (right) and the airway lumen remains stable. CT: pre-operation **(E)** and 18 months after surgery **(F)** where adequate tracheal and bronchial lumen are observed.

Figure 4 shows the images used in the clinical evaluation of a patient with BM pre- and post-operative including tracheobronchography, endoscopy and computational tomography.

#### 3.4.1 Diagnoses

A 6-month-old patient diagnosed with type I congenital tracheal stenosis with pulmonary artery sling and left bronchomalacia, that generates respiratory distress and hyperinflation of the left lung. The patient underwent cardiovascular surgery to solve the sling of the pulmonary artery but continued with symptoms and hyperinflation of the left lung. Thus, after cardiovascular surgery a study of the airway was performed, assessing it with laryngotracheobronchoscopy and tracheobronchography.

Normal larynx and normal subglottis were observed (Figure 4A). First four normal tracheal rings, then approximately 14 complete tracheal rings and last tracheal rings and carina of normal dimensions (Figure 4(A1 and A2). The tracheobronchography remarked a long congenital tracheal stenosis (more than 50% of the tracheal length), carina, and right bronchus of normal caliper without passage of contrast to the left bronchus (Figure 4A). When ventilatory pressure was applied, the left bronchus expanded and reached a good diameter, allowing the passage of contrast, confirming malacia (Figure 4B). Normal right bronchus (Figure 4C, top) and left bronchus with anterior malacia (Figure 4C, bottom), unable to identify bronchial lumen. Based on Green et al. work, this physiological abnormality might be solved by the implantation of a custom-designed and custom-fabricated resorbable airway splint (Zopf et al., 2013). This splint must provide resistance against collapse while simultaneously allowing flexion, extension, and expansion with growth. After the preoperative and intraoperative endoscopic evaluation and considering the classification of congenital tracheal stenosis presented by Grillo et al., the stenosis was classified as Type I (Sandu and Monnier, 2007). The resolution of the tracheal stenosis, as well as a slid tracheoplasty along the entire trachea and the bronchial malacia were determined. It was an allowable emergency use because the patient had a serious condition that required immediate treatment and no generally acceptable alternative treatment could be applied. Thus, the institutional Ethics Council of the clinic reviewed the case and approved the use of the splint under the emergency-use exemption. Furthermore, written informed consent was provided by the patient’s parents.

#### 3.4.2 Surgery

The surgery was performed at the Center for Medical Education and Clinical Research “Norberto Quirno” (CEMIC) on 21 August 2019, with the patient in extracorporeal circulation. A median sternotomy was performed with the patient on a pump for extracorporeal circulation. The anterior and lateral aspects of the trachea and mainstem bronchi were isolated and the left bronchomalacia was confirmed; the slide tracheoplasty was performed. After choosing the splints of best fit, one polycaprolactone exoskeleton (model 1) was placed around the left main bronchus. A series of partial thickness 4-0 polypropylene sutures were placed circumferentially around the malacic segments. The sutures were then passed through the holes of the splint, and the splint was parachuted down onto the airways. The sutures were then tied, suspending the bronchi within the splint. Surgical clips denoted proximal and distal ends of the splints for radiographic studies. Intraoperative bronchoscopy confirmed patency of the splinted regions.

#### 3.4.3 Post-operative follow-up

The patient evolved favorably and was extubated on the 6th postoperative day. A control endoscopy was performed on the 19th postoperative day, confirming good tracheal and bronchial lumen (Figure 4D, left). The patient was discharged 20 days postoperatively. The patient had no complications or intercurrences since hospital discharge. Endoscopic controls were performed at the first, third, sixth, twelfth- and 18 months post-operation. A good lumen of the airway was maintained, and the persistence of the splint was observed (Figure 4D, middle). No unforeseen problems related to the splint have arisen. At 18 months post-operation, a chest computational tomography (CT) is performed, where a good tracheal and bronchial diameter is observed. Figure 4F shows this result compared with the preoperative CT (Figure 4E). Two years and 10 months after surgery (34 months), a new endoscopy was performed, where a good tracheal and bronchial lumen was observed; no splint was observed, and the airway lumen remains stable (Figure 4D, right).

## 4 Discussion

We present the successful implantation of a bioresorbable airway splint for the management of severe BM. According to the patient’s airway anatomy and considering the standards previously established for pediatric splints (Hollister et al., 2015), the splints were made of PCL, 3D printed using FDM. FDM is a low-cost method that permits the manufacture of biocompatible and biodegradable polymers customized to the anatomical structure of the patient. According to Abedalwafa et al. (2013), PCL is a promising biomaterial for tissue engineering. It has been successfully used to produce implantable medical devices to treat serious illnesses and birth defects (Holländer et al., 2016; Siddiqui et al., 2018). PCL degradation is due to the hydrolytic cleavage of the ester groups. The time needed for complete degradation varies depending on the sample’s thickness and form; on average it takes 3 years (Sun et al., 2006), allowing for the natural regeneration of cartilage, which takes place in approximately 2 years (Jacobs et al., 1994).

Particularly, FDM is a widely available 3D printing technique that can generate medical devices on demand, automatically, without the need for tooling or other post-processing. The printer can be mounted in a clean room and remotely controlled to protect the printed structure from contamination. Each of the 3D printed splints examined in this case study may be made in less than 30 min, giving the surgeon access to multiple copies and sizes tailored to the patient in 48 h, packaging and sterilizing included. The very short time-consuming fabrication is essential when the patient’s life is in danger. It is important to note that the international regulatory organizations control the production and use of medical devices for a single patient and have established procedures for use of unapproved medical devices in patients who have no other reasonable options for treatment (Van Norman, 2018).

According to the patient airway architecture and the mechanical needs, splints designed in this study varied the internal diameter and the thickness of the printed wall (Hollister et al., 2015). Using the correct parameters for the FDM, based on the thermal characteristics of PCL and the printing pattern of each layer, excellent integration of the printed layers and a smooth surface were manufactured. It is important to note that there was no need for any post-processing. Dimensional differences between computer-aided designs and 3D-printed splints are acceptable according to the standards set by Morrison et al. (2015) for these medical devices. Furthermore, the suture hole quality was sufficient for the surgeon to attach the splint to the airway without causing any cracks or burrs.

The printing process might degrade PCL into acidic groups, identified by a broad absorption band at 3,400 cm^-1^. Also, sterilization might induce chemical changes by chain scission (Augustine et al., 2015). Nevertheless, our results show that neither FDM nor gamma irradiation to 35 kGy affect the chemical structure of PCL since the characteristic compositional peaks and bands of the FTIR for the filament and the printed splints pre- and post-irradiation exhibit no changes. Also, the melting point and degree of crystallinity were not significantly different between the PCL filament and the splints pre- and post-irradiation. Minimal differences in FTIR spectra can be attributed to crystallization processes (Phillipson et al., 2014). Culbreath et al. (2020) evaluated PLC (Caproprene 100M) after two thermal processes: extrusion and 3D printing using temperatures above 100°C. They found that the molecular weight of material remained essentially unchanged after the extrusion and 3D printing.

It is interesting to notice that implants made of PCL with similar average molecular weight became brittle after 30 months of degradation *in vivo* and could not be reclaimed at the end of 34 months after implantation. It was also reported that irradiation did not affect the adhesion and growth of chondrocytes (Cottam et al., 2009).

Besides printing quality, mechanical response of the 3D printed splints is one of the key aspects to assure a proper performance after implantation, both during breathing as well as along the growth of the airway. Forces exerted by the splint as it is deformed were measured, in compression and bending, using a dynamic mechanical analyzer. As expected, splints exhibited different mechanical behaviors for both compression and opening tests, where the most rigid model was the one with the smaller internal diameter and greater wall thickness (model 1). The dispersion between stress-strain curves was mainly due to the presence of voids inside the splint. Furthermore, the γ-irradiation dose applied for sterilization −35 kGy (Cottam et al., 2009)― did not significantly affect neither the structure nor the mechanical properties of the FDM-printed splints. Additionally, Navarro et al. demonstrated that following exposure to radiation at sterilizing levels up to 100 kGy, PCL (Mw 80000) maintained the same mechanical properties (Navarro et al., 2018). This sterilization process is strongly recommended since it is highly effective and does not leave residues, as could be the case with ethylene oxide (Naikwadi et al., 2022). Yu et al. (2022) applied a similar sterilization technique to 3D splints produced by SLS, however they do not discuss the sterilization doses or how the irradiation affected the splints’ functionality. In fact, with a median follow-up of 18 months, they showed the effectiveness of 3D printed bioresorbable external airway splints in the treatment of nine pediatric children (3–25 months) with severe TM/TBM/MB and congenital heart disease (CHD). They concluded that using 3D printed PCL splints was a trustworthy and successful treatment for those cases.

In our work, both models 1 and 2 of the 3D printed splints met the requirements for growth and maintenance of open airways, that is, the strain between plates is less than 10% under 20 N for all the samples in the compression test and at least 20% under 15 N load in opening tests (Hollister et al., 2015). Thus, the selection between models 1 and 2 was up to the surgeon´s criterion during surgery.

The pediatric patient implanted with the airway splint had a terminal form of BM. The clinical improvement was immediate and sustained after surgery. It is noteworthy that during surgery it was not necessary to add any enzyme, cell culture, or drugs at the site of implantation to enhance the efficiency to the splint function. The patient was discharged and did not require any further medication related to the implant or the BM throughout the evaluated period. This avoids the damage that can be caused to organs like the liver and the kidney by medications that prevent implant rejection. The patient has had sufficient post-operative time −34 months― to assess the splint response along the long-term airway growth.

This case shows that high-resolution imaging, computer-aided design, resorbable biomaterial, and three-dimensional printing all together can facilitate the creation of implantable devices for conditions that are anatomically specific for a given patient. The regulatory approval process and evaluation of patient candidacy needed 10 days. All devices were completed within 15 days including design and manufacturing.

Taken together, our findings suggest that 3D-printed splints produced by FDM are capable to preserve and improve the life quality of a pediatric patient diagnosed with life-threatening BM that, according to the medical criteria and the medical board involved in this case, has no other treatment options to be alive. This study remarks that FDM is an affordable 3D printing technique to produce splints that meet the requirements of similar splints produced with more sophisticated additive manufacturing devices. It does not provide any clinical guidelines, as there was no evidence of potential complications at 34 months after surgery, and only a single case is reported. Studying the properties of implantable biomedical devices manufactured by 3D printing is fundamental since they have shown high effectiveness. Furthermore, strong controls are required to understand the incidence of the manufacturing process on the acceptability of these devices.

Following van Norman´s review the splint presented in this contribution can be considered as a humanitarian device, since it was built to treat a condition affecting very small groups of patients (Van Norman, 2018). Although only one patient has been submitted to this surgical approach using a splint made by FDM, the success of this procedure represents a fundamental contribution to the treatment of the population in countries where the most expensive technologies are not available and, in many cases, involving the displacement of the patient and families, which puts the patient’s life at risk and strongly increases the costs for the families and health systems involved.

## 5 Conclusion

Splints fabricated by FDM allow in this case, to successfully surpass the breathing arrest with life-threatening BM. They are made of PCL, a biodegradable material that degrades as the airways grow and strengths until the patient breathing recovers completely. Fabrication was achieved by an affordable 3D printing technique and sterilization by gamma irradiation. Thus, this work becomes a fundamental contribution to the treatment of the population in countries where access to the most expensive technologies is not available.

## Data Availability

The original contributions presented in the study are included in the article/Supplementary Material, further inquiries can be directed to the corresponding authors.
