# Supplementary material for: Advantages of FDM and gamma irradiation to manufacture personalized medical devices for airway obstructions
Source: Front Bioeng Biotechnol. 2023 Jun 30;11:1148295. doi: 10.3389/fbioe.2023.1148295 (PMC10348745; doi:10.3389/fbioe.2023.1148295)
Supplement: Supplementary file 1 [file DataSheet1.pdf]

Supplementary Material

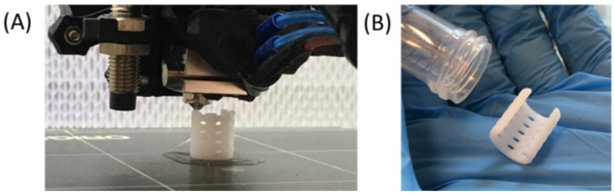

Figure S1. 3D printing process. (A) Clean area and printing bed oriented to the air flux in the horizontal laminar flux. The splint was printed onto the PCL raft. (B) PCL 3D printed splint detached from the raft.

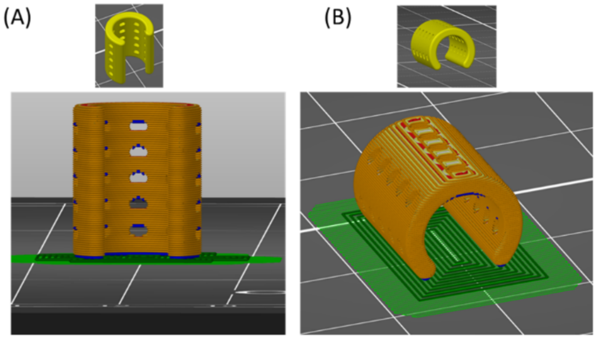

Figure S2. Splint printing orientation into the printing bed (yellow) and raft pattern design (green). (A) printing orientation as used to produce splints. (B) Splint printing orientation that produces splints with low quality.

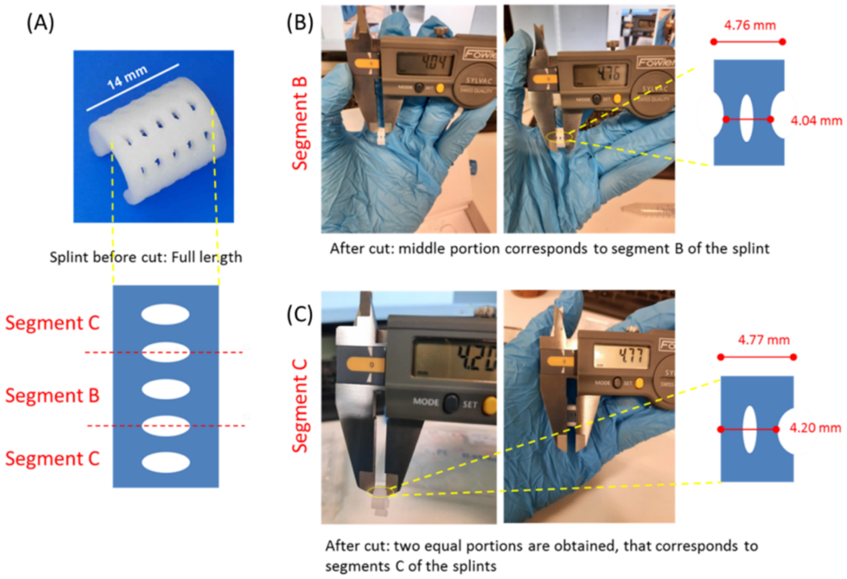

Figure S3. 3D printed splints preparation for opening tests. Splints were cut in 3 segments minimizing the strength applied to them using a cuter. Cuts were performed in between printed layers and preferably throughout the middle of suture holes, thus the segments obtained contained one set of aligned suture holes. The length of splints was ca. 14 mm. (A) splint model 1 and the scheme where the cuts were performed. (B) and (C), show the segments B and C obtained after the cut. The larger and shorter widths of segments are shown in both schemes and the distances were measured with a digital caliper.

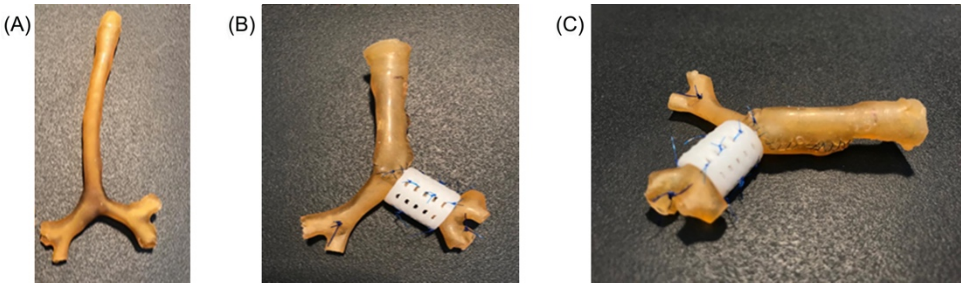

Figure S4. Model of the patient's upper airway created using SLA technology from CT scans (A). The outcome of the surgical planning is shown in the photographs; the splint is inserted onto the bronchus. In panels (B) and (C), top and lateral views, the trachea and bronchus sutures (blue lines) are seen, respectively.
